# Education in research: results of a survey commissioned by the research committee of the European Society of Radiology

**DOI:** 10.1007/s13244-012-0184-x

**Published:** 2012-09-14

**Authors:** European Society of Radiology (ESR)

**Affiliations:** European Society of Radiology (ESR), Neutorgasse 9/2, 1010 Vienna, Austria

**Keywords:** Questionnaires, Health care surveys, Education, Scientific societies, Research

## Abstract

**Objectives:**

The purpose of this investigation was to assess the current status of education in research in Europe with a view to making recommendations and improvements in the status of education in research for involved stakeholders.

**Methods:**

A questionnaire concerning education in research in Europe was sent to the National Society representatives, to Subspecialty Societies and potentially interested ESR committees. Questions were posed to assess the current status and to explore a desired future status for a broad base of interested stakeholders. Questions related to training (general status), research drivers, researcher recruitment, contents of research education, education methods, flexibility in research career planning, scientific network building, scientific research funding and measuring outcomes of research education.

**Results:**

The most pronounced inadequacies were perceived in the following areas: promotion of clinical scientists, promotion of material sciences, earlier recruitment of researchers, laboratory training, flexible and adaptable schedules, career planning by research group leaders, network building by funding agencies, funding by organ-based radiology sections and outcome measurement by professional surveys.

**Conclusions:**

The results of this questionnaire indicate that the subject of education in research methodology, career structure and career outcome need promotion. The important role of professional societies in supporting these changes is emphasised.

***Main Messages*:**

• *In the immediate future it is recommended that radiology researchers should maintain individual responsibility for their career path, but this should be actively facilitated by their peer group.*

• *A research career should be encouraged to commence during residency and include an increased proportion of wet laboratory work focussed on biologic topics.*

• *Production of peer-reviewed publications should remain a high priority.*

• *Flexibility in professional schedules should be facilitated to allow dedicated periods for formal research.*

• *Research programmes should be measured by the number of successful research-based personnel involved, in addition to the bibliography.*

## Introduction

Research is the future of radiology. It provides knowledge, innovation and visibility in the academic community, appeals to the best residents, fellows and staff radiologists, attracts industry and governmental funds, and provides data for discussions in competencies and in healthcare financing.

The proportion of radiologists’ time dedicated to research is unknown, although probably limited. Many European radiologists become involved in research after their radiology board certification, while dedicating most efforts towards clinical work during residency. Compared to other researchers, clinicians (not only radiologists) tend to be older at the time of their academic promotion.

Although research is performed at both private and academic centres, most is done at university hospitals. Unfortunately, academic institutions lose bright radiologists to better paid private practices soon after board examinations, further reducing the numbers of radiologists committed to academic and research careers.

In most countries there is increasing pressure to separate research from healthcare delivery. Hospitals often cannot afford to have residents perform research during routine working hours. External funds need to be acquired to finance such activities. Young radiologists may be tempted to aim at better paid jobs in private practices than to move into research jobs with their numerous obligations requiring longer working hours, more limited clinical experience and potentially delayed clinical promotion into clinical staff positions.

A research career should be promoted as being rewarding for the individual, building a knowledge-based and interesting radiology career with the potential of rapid academic advancement. For these reasons it is the authors’ recommendation that the European Society of Radiology become actively influential in promoting education in research. In support of this recommendation the Research Committee of the European Society of Radiology (ESR) decided to elaborate a White Paper regarding research education, which would encompass a recommended strategy for future action by ESR in this regard.

Early on, it became obvious that there is wide variability among countries and institutions regarding education in research. The decision was made to determine the status of education in research by sending a questionnaire to relevant players in order to determine the current status and the desired outcome.

The purpose of this investigation was to assess the current situation and, on this basis, make recommendations for improvement of the content, structures and delivery mechanisms for education in radiology research on a European level.

## Materials and methods

A questionnaire was designed to evaluate the status of education in research within Europe (Table 1). The questions were based on two meetings of the ESR Research Committee and a search of other sources relating to education in research, such as university programmes, ESR White Papers and radiological journals.

**Table 1:**
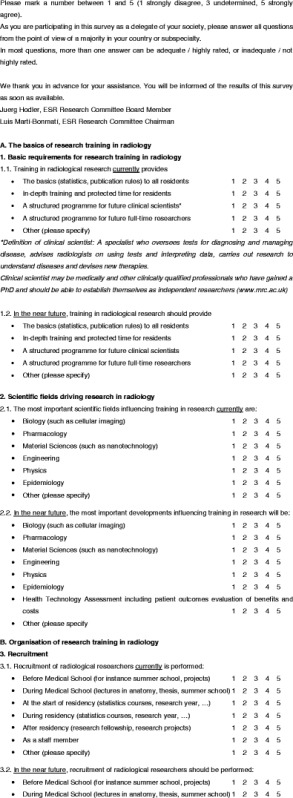

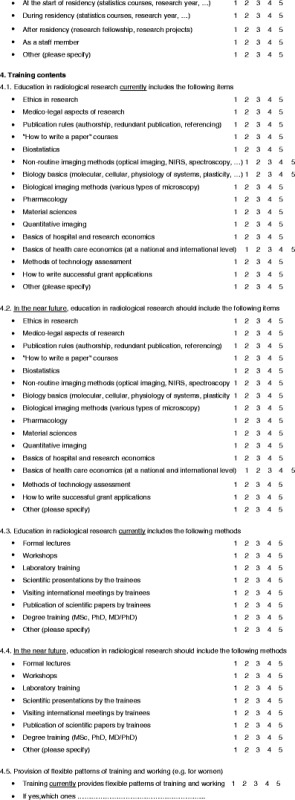

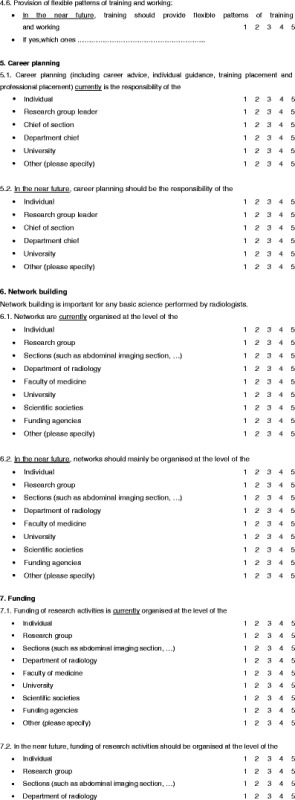

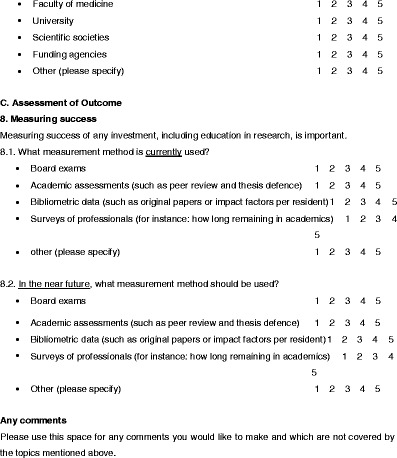
Questionnaire

The resulting questions are presented in Table 1. The same questions were asked for assessing the 2011 status in the represented countries and organisations as well as for the status that should be reached in the near future. The questions related to training (general status), research drivers, researcher recruitment, contents of research education, education methods, flexibility in research career planning, scientific network building, scientific research funding and measuring success of education. Questions could be answered with scores 1–5 (1 strongly disagree, 3 undetermined, 5 strongly agree).

The country representatives within the Research Committee, the representatives of the European subspecialty societies, the members of the Education Committee and the Health Technology Assessment Committee were invited to answer the questionnaire. The participants were asked not to act as individuals but rather to represent the point of view of a majority in their country or subspecialty.

A total of 93 representatives were contacted. An online questionnaire was produced using SurveyMonkey® online tools (http://www.surveymonkey.com/, SurveyMonkey.com, Palo Alto, CA, USA). Two reminders were sent by the ESR office in order to increase the participation.

## Results

A total of 63 questionnaires were returned.

Results are demonstrated in Tables 2 and 3. The mean mark for the questions relating to the current status was 3.12 (range: 1.79–4.11), while the mean mark for the desired status in the near future was 3.95 (range 2.27–4.55).

**Table 2:** Results

Topic	Question	Mean current	Mean future	Difference (future higher = positive values)
Training in research	The basics (statistics, publication rules) to all residents	3.13	4.43	1.30
	In-depth training and protected time for residents	2.71	4.08	1.37
	A structured programme for future clinical scientists	2.48	4.25	1.78
	A structured programme for future full-time researchers	2.40	3.84	1.44
Drivers of research	Biology (such as cellular imaging)	3.38	4.24	0.86
	Pharmacology	3.13	3.59	0.46
	Material sciences (such as nanotechnology)	2.86	3.86	1.00
	Engineering	3.38	3.65	0.26
	Physics	3.56	3.76	0.20
	Epidemiology	3.35	3.68	0.33
	Health technology assessment including patient outcomes, evaluation of benefits and costs	3.62	4.10	0.48
Recruitment	Before medical school (for instance summer school, projects)	1.79	2.27	0.48
	During medical school (lectures in anatomy, thesis, summer school)	2.75	3.77	1.02
	At the start of residency (statistics courses, research year, etc.)	2.98	4.14	1.16
	During residency (statistics courses, research year, etc.)	3.63	4.34	0.71
	After residency (research fellowship, research projects)	3.52	3.84	0.32
	As a staff member	3.38	3.39	0.02
Training contents	Ethics in research	3.02	4.27	1.25
	Medico-legal aspects of research	2.89	4.16	1.27
	Publication rules (authorship, redundant publication, referencing)	3.30	4.41	1.11
	"How to write a paper" courses	3.21	4.55	1.34
	Biostatistics	3.52	4.39	0.88
	Non-routine imaging methods (optical imaging, NIRS, spectroscopy, etc.)	2.48	3.52	1.04
	Biology basics (molecular, cellular, physiology of systems, plasticity, etc.)	2.55	3.84	1.29
	Biological imaging methods (various types of microscopy)	2.09	3.41	1.32
	Pharmacology	2.34	3.50	1.16
	Material sciences	2.13	3.36	1.23
	Quantitative imaging	2.77	4.13	1.36
	Basics of hospital and research economics	2.36	3.50	1.14
	Basics of health care economics (at a national and international level)	2.45	3.50	1.05
	Methods of technology assessment	2.73	4.09	1.36
	How to write successful grant applications	2.75	4.48	1.73
Education methods	Formal lectures	3.66	3.96	0.30
	Workshops	3.61	4.36	0.75
	Laboratory training	2.70	3.82	1.13
	Scientific presentations by the trainees	3.93	4.50	0.57
	Visiting international meetings by trainees	3.70	4.39	0.70
	Publication of scientific papers by trainees	3.71	4.55	0.84
	Degree training (MSc, PhD, MD/PhD)	3.30	4.34	1.04
Flexibility	(Women)	2.57	3.63	1.05
Responsability, career planning	Individual	4.11	4.25	0.14
	Research group leader	3.14	4.13	0.98
	Chief of section	3.14	3.86	0.71
	Department chief	3.57	3.95	0.38
	University	2.88	3.66	0.79
Network building	Individual	3.63	3.84	0.21
	Research group	3.82	4.54	0.71
	Sections (such as abdominal imaging section, etc.)	3.59	4.32	0.73
	Department of radiology	3.68	4.18	0.50
	Faculty of medicine	2.96	3.75	0.79
	University	2.79	3.55	0.77
	Scientific societies	3.71	4.34	0.63
	Funding agencies	2.48	3.29	0.80
Funding	Individual	3.27	3.32	0.05
	Research group	3.88	4.30	0.43
	Sections (such as abdominal imaging section, etc.)	2.88	3.91	1.04
	Department of radiology	3.61	4.16	0.55
	Faculty of medicine	3.09	3.80	0.71
	University	3.14	3.77	0.63
	Scientific societies	2.98	4.00	1.02
	Funding agencies	3.38	3.95	0.57
Measuring success	Board exams	3.09	3.75	0.65
	Academic assessments (such as peer review and thesis defence)	3.64	4.27	0.64
	Bibliometric data (such as original papers or impact factors per resident)	3.71	4.33	0.62
	Surveys of professionals (for instance: how long remaining in academics)	2.80	3.65	0.85
Mean values		3.12	3.95	0.83

**Table 3:** Most relevant points

Topic	Current highest grade	Desired highest grade	Largest difference between current and desired status
Training in research	Basics (statistics, etc.)	Basics (statistics, etc.)	Programmes for clinical scientists
Drivers of research	Health technology assessment	Biology	Material sciences
Recruitment	During and after residency	During residency	Start of residency
Training contents	Biostatistics	How to write a paper	Grant applications
Education methods	Presentations given by trainees	Publications by trainees	Laboratory training
Flexibility	Below average	Above average	Should become better
Responsability, career planning	Individual researcher	Individual researcher	Research groups
Network building	Research group	Research group	Funding agencies and faculty of medicine
Funding	Research group	Research group	Organ-based radiology sections and scientific societies
Measuring success	Bibliometric data	Professional outcome and academic assessments	Professional outcome

Regarding the current training in research issues, the most commonly employed type is teaching of basics (such as statistics and publication rules) to all residents (mean mark 3.11). The currently most pronounced driver of research is considered to be health technology assessment (HTA, mean mark 3.62) and physics (3.56). Researchers are typically recruited during (3.63) and after (3.56) residency. The most prominent content of training in research is biostatistics (3.52), and the most commonly employed teaching method is presentations given by trainees (3.93). Flexibility of training in research is rated below average (2.57). Career planning is typically done by the researchers themselves (4.11), network building by the research groups (3.82) and funding by research groups (3.88). Success is measured by bibliometric data (3.71).

When asked about the desired future, training in the basics remains on top (4.43). Biology (such as cellular imaging) is considered to be the most pronounced driver of research in radiology (4.24). Recruitment of researchers should occur during residency (4.34). "How to write a paper" courses were considered to represent the most important contents of research training (4.55). The most desirable education is hands-on training, consisting of publication of original papers by residents (4.55). Career planning is considered to best remain in the hands of the individual (4.25), network building and funding in the hands of research groups (4.54 and 4.30, respectively). Bibliometric data remain the most accepted form of outcome measurement (4.33), closely followed by academic assessments (4.27).

The difference between current marks and marks for the desired future may indicate where the responsible persons and institutions should be most active. With regard to training in general, the largest gap between the current status and desired future was found with regard to structured programmes for clinical scientists (current availability marked as 2.48, desired status as 4.25, resulting in a difference of +1.78 after rounding). When looking at drivers of research in radiology, material sciences are considered to be underrepresented (current: 2.86, desired future: 3.86, difference of +1.00). With regard to recruitment, emphasis should most typically be placed at the start of the residency (currently 2.98, desired status 4.14, resulting in a difference of +1.16). Changes in training contents should increasingly emphasise grant applications (currently 2.75, desired future 4.48, resulting in a gap of +1.73). With regard to education methods, laboratory training should become more important (currently 2.70, desired future status 3.82, resulting in a gap of +1.13 after rounding). Training programmes should become far more flexible (current availability 2.57, desired future 3.63, resulting in a gap of +1.05 after rounding). Research group leaders should take over more responsibility for career planning (currently 3.14, desired future 4.13, resulting in a gap of +0.98). Funding agencies should become more active in network building (currently 2.48, desired status 3.29, gap +0.80, closely followed by faculties of medicine and universities). Funding should increasingly be sought by organ-based radiology sections (such as abdominal imaging) (currently 2.88, desired status 3.91, gap of +1.04), closely followed by scientific societies (+1.02). Success of the training programme should more often be measured by an outcome survey of professionals (currently 2.80, desired future 3.65, gap of +0.85).

Open comments included descriptions of country-specific or specialty society-centred activities such as board’s exams, national centralised courses, industry-sponsored courses and lectures at national society meetings. There were comments that funding by educational versus healthcare agencies may represent a problem for research education. Also, there were fears of disintegration of radiology departments caused by different careers for clinical and research residents.

## Discussion

There is no doubt that research is the future of any clinical discipline, including radiology. At the same time, many countries find it difficult to fund research adequately and many individuals may not look at a research career as a rewarding opportunity. There is no reason to sit back because many European initiatives are very successful, including the ESR and its subspecialty societies, institutions and programmes including ESOR (European School of Radiology) and EIBIR (European Institute for Biomedical Imaging Research). The purpose of this investigation was to assess the current as well as the desired status of education in research in Europe in order to be able to guide future activities of the involved stakeholders.

Before this initiative, there have been a number of White Papers published by the ESR making partial statements about the role of research in education. However, this was only one of many aspects presented in these papers. The White Paper on Imaging Biomarkers [1] asks for research fellowship programmes in quantitative imaging and biomarkers. An ESR position paper about Research in Cardiac Imaging looks for a structured mentoring programme, an increase in radiologist’s activities in basic cardiac imaging research, exposure of all residents to research training, including the knowledge of study design, methods, data management and statistics, public recognition of research efforts by residents, funding of research and scientific meeting attendance [2]. A positional paper by Sardanelli et al. [3] about Evidence-Based Radiology asks for inclusion of evidence-based medicine principles including biostatistics into the residency programme and for quantification the level of involvement of residents in radiology in radiological research, for promotion of courses by the ESOR. A planned White Paper in Molecular Imaging assumes that residency programmes will change and that there should be grants for research in molecular imaging (presented at the Research Committee Meeting of the ESR, Vienna, 2012).

The European Training Charter also stated that knowledge of basic elements of scientific methods and evidence-based training, including basic knowledge statistics necessary for critical assessment and understanding of published papers. The Training Charter also recommends the promotion of personal research and that a dedicated period of research of up to 1 year should be permissible as part of the overall training programme. Trainees should be encouraged to undertake a research project during their training, and this is particularly valid during the years of subspecialty interest training [4].

Based on a questionnaire distributed at the ENCITE (European Network for Cell Imaging and Tracking Expertise) meetings of EIBIR research leaders, there is a need to guide upcoming scientists and to teach skills such as project management, writing of publications and grants; teamwork should also be included in the training (personal communication by the EIBIR office).

Our own questionnaire did not directly aim at research-oriented institutions, but rather at the national societies and subspecialty societies, which are closer to the realities of clinical work and the political situation.

The representatives see research in radiology as follows (Table 3): Education in research is currently mostly based on the basics of research. The most important driver of research is considered to be health technology assessment. Researchers are recruited rather late (during or even after their residency). The most important topic within the training programmes is biostatistics. Trainees are typically exposed to presentations as a training method. There is a lack of flexible training programmes, which may for instance allow women to proceed with their career when families have young children. The individual is currently primarily responsible for his or her own career. The research group typically provides network building and funding, and success is mostly measured by bibliometric data. This is probably not the model of the future (the late-coming researcher without a family, without professional guidance, assessing developments others have driven and mostly presenting them at meetings instead of producing original articles).

The ideal of the near future would still be a researcher organising his own career but starting earlier, during residency, probable doing wet laboratory work, dealing with biological topics and producing peer-reviewed papers, all this in combination with a normal family life. To recruit researchers early is of importance because early involvement more often leads to an academic career [5]. Programmes might be measured by the number of successful careers rather than simply by bibliometry, for instance by surveys about the professional outcome of former trainees.

The biggest gap between the current status and the desired future, however, has been identified as follows: Radiology should provide programmes for clinical scientists, invest far more in material science programmes, more commonly recruit early (at the beginning of the residency programme), send residents to laboratories more often, provide more guidance for career planning and increase the number of stakeholders contributing to network building and funding (funding agencies, faculties of medicine, radiology sections and scientific societies). There is quite a large perceived gap between the current status of outcome measurement and the desired status.

The questionnaire did not include all stakeholders. Governments, health insurance companies, patients and patient organisations as well as the private sector may have quite different ideas. However, from the point of view of a professional society such as the ESR, the most important players have been included.

There may be comments that radiology performs far better than indicated in this article and that a fair amount of improvement has already been reached. However, we should not only look at ourselves but benchmark with other medical disciplines such as internal medicine and its subspecialties (personal communication, Gary Glazer, Stanford University).

One interesting point of this survey is the emphasis that should be placed on clinical scientists. Clinical scientists may be defined as those individuals holding an MD or MD-PhD degree that perform biomedical research of any type as their primary professional activity [6]. MD-PhD programmes of European universities fit into this concept.

Many aspects of this questionnaire have a direct relationship with professional societies. Although they have to concentrate on a large number of tasks and have limited resources, they can influence the future of radiology by means of education in research, for instance by providing training during meetings and courses such as those of the ESOR, providing guidance for residency programmes by adapting the training charter and other guidelines, by assisting research groups with direct funding or assistance in funding, such as the EIBIR does for European grants.

In conclusion, the results of this questionnaire indicate a wide variability across Europe in approaches and programmes for education in radiology research. It is recommended that education in radiology research should be promoted. The advantages of dedicated time at an early stage in radiology training should be highlighted, and a specific career structure for education in research should be elaborated and recommended. The important role of the national and subspecialty societies in this regard is acknowledged.

## References

[1] European Society of Radiology (ESR) (2010). White paper on imaging biomarkers. Insights Imaging.

[2] ESR Working Group on Cardiac Imaging Research of the European Society of Cardiac Radiology (2010). Research in cardiac radiology: a European Society of Radiology white paper. Insights Imaging.

[3] Sardanelli F, Hunink MG, Gilbert FJ, Di Leo G, Krestin GP (2010). Evidence-based radiology: why and how?. Europ Radiol.

[4] Revised European Training Charter for Clinical Radiology, http://www.myesr.org/html/img/pool/EuropeanTrainingCharter_current_version_May_2012.pdf 10.1007/s13244-011-0143-y PMC3292641 22695993

[5] Hillman BJ, Witzke DB, Fajardo LL, Fulginiti JV (1990). Research and research training in academic radiology departments. A survey of department chairmen. Invest Radiol.

[6] Zemlo TR, Garrison HH, Partridge NC, Ley TJ (2000). The physician-scientist: career issues and challenges at the year 2000. FASEB J.

